# Longitudinal vibration control of a double-rod system by employing nonlinear energy sinks

**DOI:** 10.1038/s41598-024-59644-w

**Published:** 2024-04-20

**Authors:** Yuhao Zhao, Zheng Li, Haijian Cui, Deshui Xu

**Affiliations:** 1 Key Laboratory of Advanced Manufacturing Technology of the Ministry of Education, Guizhou University, Guiyang, 550025 People’s Republic of China; 2 Wuhan Second Ship Design and Research Institute, Wuhan, 430064 People’s Republic of China; 3 Nuclear Power Institute of China, Chengdu, 610005 People’s Republic of China

**Keywords:** Longitudinal vibration, Double-rod system, Nonlinear energy sink, Galerkin method, Mechanical engineering, Civil engineering

## Abstract

This study aims to potential the potential utilization of nonlinear energy sinks (NESs) for controlling longitudinal vibrations in a double-rod system. The research introduces a longitudinal vibration prediction model for a double-rod system equipped with NESs. The generalized Hamilton principle is employed to derive governing equations of the double-rod system. The longitudinal vibration responses of the double-rod system are numerically solved through the application of Galerkin truncation method. The longitudinal vibration responses of the double-rod system are impacted by NESs, as they yield accurate numerical results. The installation of both NES 1 and NES 2 concurrently is recommended for mitigating the vibration of the double-rod system. Under reasonable single-frequency excitations, modifying the parameters of NESs can significantly alter both the vibration state and magnitudes of vibration in the double-rod system. Furthermore, the synchronous optimization of parameters in NES 1 and NES 2 is crucial for effectively controlling vibrations in the double-rod system. Sensitive parameter areas of NESs provide the possibility of controlling the vibration of the double-rod system by utilizing NESs.

## Introduction

During numerous engineering scenarios, vibration issues caused by power machinery are readily noticeable in intricate structures comprising rods, beams, and other components. In marine engineering, the shafting system serves as a pertinent example. This system can be delineated as comprising multiple rods that endure longitudinal thrust emanating from the propeller. The excitation frequency of longitudinal thrust is commonly dictated by the rotational speed of propellers. The presence of longitudinal excitations can lead to longitudinal vibration in the shafting system. The occurrence of unforeseen longitudinal vibrations in the shafting system may lead to significant safety concerns. To mitigate the vibration in shafting systems, it is essential to conduct an in-depth investigation into the vibration characteristics of the rod system.

Candan and Elishakoff^1^ investigated the longitudinal vibration mode shapes of a rod system composed of elastic structures. Erol^2^ derived and resolved the longitudinal vibration characteristic equations of the rod system incorporating multiple spring-mass systems. Davey and Rasgado^3^ proposed an analytical solution for addressing vibration issues in composite rod systems. Mei^4^ examined various rod theories and conducted a comparative analysis of their strengths and weaknesses, providing valuable insights for selecting rod theories in engineering applications. Goldberg and O'Reilly^5^ conducted a study on the effect of contact point motion on the vibration characteristics of rods. Xu et al.^6^ utilized an enhanced Fourier series in conjunction with the energy principle to investigate the longitudinal vibration properties of the nonlocal rod system. The aforementioned literature primarily relies on the linear vibration theory. Upon examination of the rod system, researchers have discovered that nonlinear vibration within the rod system can be induced under specific circumstances. Cao and Tucker^7^ employed Cosserat theory to conceptualize and simulate the nonlinear dynamics of the rod system. Wang and Li^8^ developed a vibration prediction model for a double-rod system connected by a clearance joint. Andrianov et al.^9^ investigated nonlinear vibrations and mode interactions in a rod system with microstructure. Malara et al.^10^ introduced an approximate analytical solution utilizing the statistical linearization technique to investigate the nonlinear stochastic vibration of a rod with a variable cross-section. Huang et al.^11^ proposed a modified restoring force model for reinforced concrete (RC) columns and subsequently investigated the seismic performance of twenty-five such columns. Zhang et al.^12^ used an innovative stochastic homotopy method for analyzing the static response of structures with random variables following arbitrary distributions. This method aims to minimize the stochastic residual error.

With advancements in vibration control technology, engineers have started incorporating nonlinearities in the design of equipment to manage the vibration of elastic structures. Recently, nonlinear equipment has found extensive application across diverse engineering domains such as aerospace equipment vibration control, ship vibration reduction, and other related areas. Lu et al.^13,14^ proposed a two-stage nonlinear vibration isolation system and conducted a comprehensive investigation into the vibration isolation of vibrating structures using the aforementioned isolation system. Hao et al.^15^ developed and investigated the shock isolation capabilities of an orthogonal six-degrees-of-freedom platform with high static stiffness and low dynamic stiffness. Furthermore, to expand the utilization of nonlinearities, researchers have introduced and examined NESs^16–18^. In light of this context, researchers endeavored to implement NESs on flexible structures to regulate their vibration. Georgiades and Vakakis^19^ implemented a local NES on a linear beam to regulate its vibration amplitude. Ahmadabadi and Khadem^20^ installed NES in a cantilever beam, offering theoretical backing for nonlinear vibration control. Kani et al.^21^ and Bukhari and Barry^22^ investigated the nonlinear behavior of a beam connected to a NES, considering various boundary conditions applied to the beam system. Kani et al.^23^ deployed a NES on a nonlinear beam and examined the control of nonlinear vibrations in the beam system through the utilization of NES. Chen et al.^24^ utilized parallel NESs to mitigate the vibration of the beam system, introducing a novel approach to beam vibration control. He et al.^25^ initially developed a vibration prediction model for the beam by integrating the acoustic black hole and NES. This model aimed to mitigate beam vibration across the entire frequency spectrum. Moslemi et al.^26^ employed NES to mitigate the nonlinear vibration of a beam in axial motion. Zhang et al.^27,28^ utilized the boundary inerter-enhanced NES method to manage the vibration of both linear and nonlinear beams. Wang et al.^29^ used NES method to mitigate the vibration of a cable-stayed beam. Chang et al.^30^ employed the inertial NES to attenuate the undesired vibrations in a nonlinear beam structure. Cao et al.^31^ installed a non-smooth NES into the rotor-blade system, thereby advancing the utilization of NESs. Zhang et al.^32^ incorporated NES into a composite laminated plate to investigate the potential for vibration control in such plates through the utilization of NESs. Zhang and Chen^33^ endeavored to employ NES for the purpose of managing the vibration of a rectangular plate. Yao and Qiao^34^ explored the utilization of an energy harvester-enhanced NES for the absorption and mitigation of plate vibrations. Zhang et al.^35^ utilized internal oscillator-enhanced negative stiffness elements to mitigate the vibration of nonlinear laminated composite plates. The existing researches primarily focused on exploring the potential utilization of NES devices in elastic structures, such as beams and plates, which are commonly considered as individual structural elements. Limited scholarly works have explored the potential utilization of NES for vibration control in systems consisting of multiple components. Furthermore, the predominant focus of existing studies lies in examining the performance of individual NES units. Consequently, the potential benefits and challenges associated with employing multiple NESs for structural vibration control have not been comprehensively explored. This knowledge gap hinders the widespread adoption of NESs for vibration control in intricate structural systems.

Given the aforementioned limitations, this study proposes a predictive model for longitudinal vibration in a double-rod system with NES devices. The generalized Hamilton principle is utilized to deduce the governing equations of the double-rod system. The longitudinal vibration responses of the double-rod system are subsequently determined through numerical solutions employing GTM. After guaranteeing the validity of the numerical results, longitudinal vibration responses of the double-rod system affected by NESs are discussed.

## Theoretical formulations

### Model description

**Figure 1:**
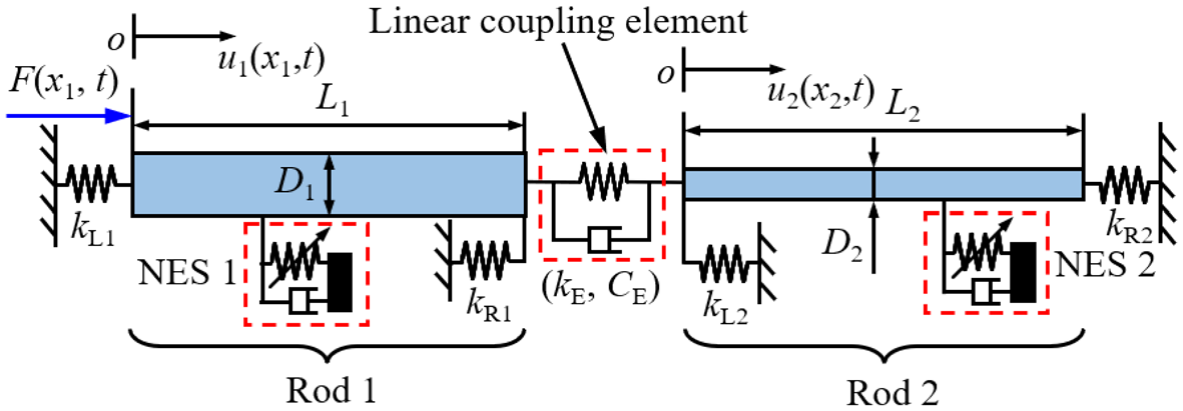
Schematic diagram of a generally constrained double-rod system attached with NESs.

Figure 1 presents the diagram of a double-rod system with NESs. The double-rod system is composed of Rod 1 and Rod 2, where Rod 1 and Rod 2 are connected through a linear coupling element. The linear coupling element is simplified as longitudinal linear stiffness and external viscous damping. Longitudinal-constrained springs are installed at the boundaries of each rod, which are used to provide longitudinal supporting stiffness. The longitudinal harmonic excitation is located on the left end of Rod 1, which motivates the longitudinal vibration of the double-rod system. NES 1 is installed on Rod 1 while NES 2 is installed on Rod 2, where *x*_NES1_ is the installation location of NES 1 while *x*_NES2_ is that of NES 2. To distinguish NES 1 and NES 2, *m*_NES1_, *m*_NES2_, *k*_NES1_, *k*_NES2_, *C*_NES1_, and* C*_NES2_ are defined as the motion mass, nonlinear stiffness, and external viscous damping of NES1 and NES 2, respectively. Table 1 gives symbol definitions of the double-rod system.

**Table 1:** Symbol definitions of the double-rod system.

Parameters	Symbol	Unit
Elastic modulus of rods	*E*_1_/*E*_2_	Pa
Density of rods	*ρ*_1_/*ρ*_2_	kg/m^3^
Length of rods	*L*_1_/*L*_2_	m
Diameter of rods	*D*_1_/*D*_2_	m
Cross area	*S*_1_/*S*_2_	m^2^
Longitudinal-constrained springs	*k*_L1_/*k*_L2_/*k*_R1_/*k*_R2_	N/m
Stiffness of the linear coupling element	*k*_E_	N/m
Viscous damping of the linear coupling element	*C*_E_	Ns/m
The magnitude of the longitudinal excitation	*F*_E_	N
Angle frequency of the longitudinal excitation	*ω*	Rad/s

Compared with linear tuned mass damper (TMD), in practical engineering, structural fatigue, degradation, and dimensional changes may lead to changes in structural stiffness or natural frequency, which can cause TMD to lose tuning and result in low efficiency. In addition, external excitation in practical engineering may be very complex, and the excitation characteristics may change. Installing TMD can lead to the formation of new resonance peaks on both sides of the tuning frequency. When the external excitation frequency changes, it may even exacerbate the vibration of the original structure. It is worth mentioning that the nonlinear stiffness of NESs presents the cubic stiffness characteristic in this work and does not have a fixed natural frequency, which can generate resonance energy capture for vibrations at any frequency. Thus, this work studies longitudinal vibration of a double-rod system with NESs.

### Equations derivation

In this work, the generalized Hamilton principle is used to derive governing equations of the double-rod system with NESs. It should be noticed that gaining the form of vibration energy belonging to the double-rod system with NESs is the basement of deriving governing equations by employing the generalized Hamilton principle. The vibration energy of the double-rod system with NESs consists of potential energy and kinetic energy. According to the energy principle, the potential energy of the double-rod system with NESs is derived as,

1 $$V_{{{\text{System}}}} = V_{{{\text{Rod1}}}} + V_{{{\text{Rod2}}}} + V_{{{\text{Boundary1}}}} + V_{{{\text{Boundary2}}}} + V_{{{\text{LE}}}} + V_{{{\text{NES1}}}} + V_{{{\text{NES2}}}}$$

*V*_Rod1_ and *V*_Rod2_ are the potential energy of rods. *V*_Boundary1_ and *V*_Boundary2_ are the potential energy of constrained springs located at rods. *V*_LE_ is the potential energy of the linear coupling element. *V*_NES1_ and *V*_NES2_ are the potential energy of NESs.

The kinetic energy of the double-rod system with NESs is derived as,

2 $$T_{{{\text{System}}}} = T_{{{\text{Rod1}}}} + T_{{{\text{Rod2}}}} + T_{{{\text{NES1}}}} + T_{{{\text{NES2}}}}$$

*T*_Rod1_ and *T*_Rod2_ are the kinetic energy of rods. *T*_NES1_ and *T*_NES2_ are the kinetic energy of NESs. The virtual work working on the double-rod system is derived as,

3 $$\delta W_{{{\text{System}}}} = \delta W_{{\text{E}}} + \delta W_{{{\text{LE}}}} + \delta W_{{{\text{NES1}}}} + \delta W_{{{\text{NES2}}}}$$

*δW*_E_ is the virtual work made by harmonic excitation. *δW*_LE_ is the virtual work made by viscous damping of the linear coupling element. *δW*_NES1_ *and δW*_NES2_ are the virtual work made by viscous damping of NESs. Specific expressions of terms in Eqs. (1–3) are listed in Appendix A.

On this basis, employ the generalized Hamilton principle, namely,

4 $$\int_{{t_{1} }}^{{t_{2} }} {\delta \left( {T_{{{\text{System}}}} - V_{{{\text{System}}}} } \right){\text{d}}t} + \int_{{t_{1} }}^{{t_{2} }} {\delta W_{{{\text{System}}}} {\text{d}}t} = 0$$

Then, the variational step is to derive governing equations of the double-rod system with NESs. Vibration-governing equations of rods are derived as,

5-a $$\begin{gathered} \begin{array}{*{20}c} {} & {\rho_{1} S_{1} \frac{{\partial^{2} u_{1} }}{{\partial t^{2} }} - E_{1} S_{1} \frac{{\partial^{2} u_{1} }}{{\partial x_{1}^{2} }} + \delta \left( {x_{1} - L_{1} } \right)\delta \left( {x_{2} } \right)\left[ {k_{{\text{E}}} \left( {u_{1} - u_{2} } \right) + C_{{\text{E}}} \left( {\frac{{\partial u_{1} }}{\partial t} - \frac{{\partial u_{2} }}{\partial t}} \right)} \right]} \\ \end{array} \hfill \\ + \delta \left( {x_{1} - x_{{{\text{NES1}}}} } \right)\left[ {k_{{{\text{NES1}}}} \left( {u_{1} - u_{{{\text{NES1}}}} } \right)^{3} + C_{{{\text{NES1}}}} \left( {\frac{{\partial u_{1} }}{\partial t} - \frac{{{\text{d}}u_{{{\text{NES1}}}} }}{{{\text{d}}t}}} \right)} \right] + \delta \left( {x_{1} } \right)F_{{\text{E}}} \sin \left( {\omega t} \right) = 0 \hfill \\ \end{gathered}$$

and

5-b $$\begin{gathered} \rho_{2} S_{2} \frac{{\partial^{2} u_{2} }}{{\partial t^{2} }} - E_{2} S_{2} \frac{{\partial^{2} u_{2} }}{{\partial x_{2}^{2} }} + \delta \left( {x_{1} - L_{1} } \right)\delta \left( {x_{2} } \right)\left[ {k_{{\text{E}}} \left( {u_{2} - u_{1} } \right) + C_{{\text{E}}} \left( {\frac{{\partial u_{2} }}{\partial t} - \frac{{\partial u_{1} }}{\partial t}} \right)} \right] \hfill \\ \begin{array}{*{20}c} {} & {} \\ \end{array} + \delta \left( {x_{2} - x_{{{\text{NES2}}}} } \right)\left[ {k_{{{\text{NES2}}}} \left( {u_{2} - u_{{{\text{NES2}}}} } \right)^{3} + C_{{{\text{NES2}}}} \left( {\frac{{\partial u_{2} }}{\partial t} - \frac{{{\text{d}}u_{{{\text{NES2}}}} }}{{{\text{d}}t}}} \right)} \right] = 0 \hfill \\ \end{gathered}$$

where *u*_1_(*x*_1_,*t*) and *u*_2_(*x*_2_,*t*) are longitudinal displacements of rods. *u*_NES1_(*t*) and *u*_NES2_(*t*) are vibration displacements of NESs. Equation (5-a) is the vibration-governing equation of Rod 1 while Eq. (5-b) is the vibration-governing equation of Rod 2. Boundary-governing equations of rods are derived as,

6-a $$\left\{ \begin{gathered} k_{{{\text{L1}}}} u_{1} - E_{1} S_{1} \frac{{\partial u_{1} }}{{\partial x_{1} }} = 0\begin{array}{*{20}c} , \\ \end{array} \begin{array}{*{20}c} {} \\ \end{array} x_{1} = 0 \hfill \\ k_{{{\text{R1}}}} u_{1} + E_{1} S_{1} \frac{{\partial u_{1} }}{{\partial x_{1} }} = 0\begin{array}{*{20}c} , \\ \end{array} \begin{array}{*{20}c} {} \\ \end{array} x_{1} = L_{1} \hfill \\ \end{gathered} \right.$$

and

6-b $$\left\{ \begin{gathered} k_{{{\text{L2}}}} u_{2} - E_{2} S_{2} \frac{{\partial u_{2} }}{{\partial x_{2} }} = 0\begin{array}{*{20}c} , \\ \end{array} \begin{array}{*{20}c} {} \\ \end{array} x_{2} = 0 \hfill \\ k_{{{\text{R2}}}} u_{2} + E_{2} S_{2} \frac{{\partial u_{2} }}{{\partial x_{2} }} = 0\begin{array}{*{20}c} , \\ \end{array} \begin{array}{*{20}c} {} \\ \end{array} x_{2} = L_{2} \hfill \\ \end{gathered} \right.$$

Equation (6-a) is the boundary-governing equation of Rod 1 while Eq. (6-b) is the boundary-governing equation of Rod 2. Motion equations of NESs are derived as,

7-a $$m_{{{\text{NES1}}}} \frac{{{\text{d}}^{2} u_{{{\text{NES1}}}} }}{{{\text{d}}t^{2} }} + \delta \left( {x_{1} - x_{{{\text{NES1}}}} } \right)\left[ {k_{{{\text{NES1}}}} \left( {u_{{{\text{NES1}}}} - u_{1} } \right)^{3} + C_{{{\text{NES1}}}} \left( {\frac{{{\text{d}}u_{{{\text{NES1}}}} }}{{{\text{d}}t}} - \frac{{\partial u_{1} }}{\partial t}} \right)} \right] = 0$$

and

7-b $$m_{{{\text{NES2}}}} \frac{{{\text{d}}^{2} u_{{{\text{NES2}}}} }}{{{\text{d}}t^{2} }} + \delta \left( {x_{2} - x_{{{\text{NES2}}}} } \right)\left[ {k_{{{\text{NES2}}}} \left( {u_{{{\text{NES2}}}} - u_{2} } \right)^{3} + C_{{{\text{NES2}}}} \left( {\frac{{{\text{d}}u_{{{\text{NES2}}}} }}{{{\text{d}}t}} - \frac{{\partial u_{2} }}{\partial t}} \right)} \right] = 0$$

Obviously, based on the above derivation, dynamic responses of the double-rod system with NESs can be predicted by solving Eqs. (5–7). The detailed derivation procedure of the variational step is given in Appendix B.

### Procedure of solution

Based on the governing equations derived in Section “Equations derivation”, this section concentrates on their solution procedure. Obviously, the key to obtaining dynamic responses of the double-rod system with NESs is to discrete vibration-governing equations of rods. In this work, the Galerkin condition is used to discrete the corresponding equations. Longitudinal displacements of rods are assumed as the form of mode superposition, namely,

8-a $$u_{1} \left( {x_{1} ,t} \right) = \sum\limits_{n = 1}^{{\text{N}}} {\varphi_{1n} \left( {x_{1} } \right)q_{1n} \left( t \right)}$$

and

8-b $$u_{2} \left( {x_{2} ,t} \right) = \sum\limits_{m = 1}^{{\text{M}}} {\varphi_{2m} \left( {x_{2} } \right)q_{2m} \left( t \right)}$$

*φ*_1*n*_(*x*_1_) and *φ*_2*m*_(*x*_2_) are the mode functions of rods. *q*_1*n*_(*t*) and *q*_2*m*_(*t*) are the undetermined coefficients. N and M are the truncation number. Substituting Eq. (8) into Eq. (5) and using the Galerkin condition, vibration-governing equations of rods are dispersed as,

9-a $$\begin{gathered} \int_{0}^{{L_{1} }} {\left( {\rho_{1} S_{1} \sum\limits_{n = 1}^{{\text{N}}} {\varphi_{1n} \frac{{{\text{d}}^{2} q_{1n} }}{{{\text{d}}t^{2} }}} - E_{1} S_{1} \sum\limits_{n = 1}^{{\text{N}}} {\frac{{{\text{d}}^{2} \varphi_{1n} }}{{{\text{d}}x_{1}^{2} }}q_{1n} } } \right)\psi_{1i} \left( {x_{1} } \right){\text{d}}x_{1} } \hfill \\ + F_{{\text{E}}} \sin \left( {\omega t} \right)\left[ {\sum\limits_{n = 1}^{{\text{N}}} {\varphi_{1n} \left( 0 \right)q_{1n} } } \right]\psi_{1i} \left( 0 \right) \hfill \\ + k_{{\text{E}}} \left[ {\sum\limits_{n = 1}^{{\text{N}}} {\varphi_{1n} \left( {L_{1} } \right)q_{1n} } - \sum\limits_{m = 1}^{{\text{M}}} {\varphi_{2m} \left( 0 \right)q_{2m} } } \right]\psi_{1i} \left( {L_{1} } \right) \hfill \\ + C_{{\text{E}}} \left[ {\sum\limits_{n = 1}^{{\text{N}}} {\varphi_{1n} \left( {L_{1} } \right)\frac{{{\text{d}}q_{1n} }}{{{\text{d}}t}}} - \sum\limits_{m = 1}^{{\text{M}}} {\varphi_{2m} \left( 0 \right)\frac{{{\text{d}}q_{2m} }}{{{\text{d}}t}}} } \right]\psi_{1i} \left( {L_{1} } \right) \hfill \\ + k_{{{\text{NES1}}}} \left[ {\sum\limits_{n = 1}^{{\text{N}}} {\varphi_{1n} \left( {x_{{{\text{NES1}}}} } \right)\frac{{{\text{d}}q_{2n} }}{{{\text{d}}t}}} - u_{{{\text{NES1}}}} } \right]^{3} \psi_{1i} \left( {x_{{{\text{NES1}}}} } \right) \hfill \\ + C_{{{\text{NES1}}}} \left[ {\sum\limits_{n = 1}^{{\text{N}}} {\varphi_{1n} \left( {x_{{{\text{NES1}}}} } \right)\frac{{{\text{d}}q_{1n} }}{{{\text{d}}t}}} - \frac{{{\text{d}}u_{{{\text{NES1}}}} }}{{{\text{d}}t}}} \right]\psi_{1i} \left( {x_{{{\text{NES1}}}} } \right) = 0 \hfill \\ \end{gathered}$$

and

9-b $$\begin{gathered} \int_{0}^{{L_{2} }} {\left( {\rho_{2} S_{2} \sum\limits_{m = 1}^{{\text{M}}} {\varphi_{2m} \frac{{{\text{d}}^{2} q_{2m} }}{{{\text{d}}t^{2} }}} - E_{2} S_{2} \sum\limits_{m = 1}^{{\text{M}}} {\frac{{{\text{d}}^{2} \varphi_{2m} }}{{{\text{d}}x_{2}^{2} }}q_{2m} } } \right)\psi_{2j} \left( {x_{2} } \right){\text{d}}x_{2} } \hfill \\ + k_{{\text{E}}} \left[ {\sum\limits_{m = 1}^{{\text{M}}} {\varphi_{2m} \left( 0 \right)q_{2m} } - \sum\limits_{n = 1}^{{\text{N}}} {\varphi_{1n} \left( {L_{1} } \right)q_{1n} } } \right]\psi_{2j} \left( 0 \right) \hfill \\ + C_{{\text{E}}} \left[ {\sum\limits_{m = 1}^{{\text{M}}} {\varphi_{2m} \left( 0 \right)\frac{{{\text{d}}q_{2m} }}{{{\text{d}}t}}} - \sum\limits_{n = 1}^{{\text{N}}} {\varphi_{1n} \left( {L_{1} } \right)\frac{{{\text{d}}q_{1n} }}{{{\text{d}}t}}} } \right]\psi_{2j} \left( 0 \right) \hfill \\ + k_{{{\text{NES2}}}} \left[ {\sum\limits_{m = 1}^{{\text{M}}} {\varphi_{2m} \left( {x_{{{\text{NES2}}}} } \right)\frac{{{\text{d}}q_{2m} }}{{{\text{d}}t}}} - u_{{{\text{NES2}}}} } \right]^{3} \psi_{2j} \left( {x_{{{\text{NES2}}}} } \right) \hfill \\ + C_{{{\text{NES2}}}} \left[ {\sum\limits_{m = 1}^{{\text{M}}} {\varphi_{2m} \left( {x_{{{\text{NES2}}}} } \right)\frac{{{\text{d}}q_{2m} }}{{{\text{d}}t}}} - \frac{{{\text{d}}u_{{{\text{NES2}}}} }}{{{\text{d}}t}}} \right]\psi_{2j} \left( {x_{{{\text{NES2}}}} } \right) = 0 \hfill \\ \end{gathered}$$

To ensure the number of equations equal to the number of undetermined coefficients, the max value of *i* is N while that of *j* is M. *ψ*_1*i*_(*x*_1_) and *ψ*_2*j*_(*x*_2_) are called the weight functions, which should meet boundary-governing equations of the vibration system. Mode functions of the single rod meet Eqs. (6-a) and (6-b), which are chosen as the weight functions^27^. The above procedure based on the modal information of rods is called the Galerkin truncation method.

Then, substituting Eq. (8) into Eq. (7), motion equations of NESs are rewritten as,

10-a $$\begin{gathered} m_{{{\text{NES1}}}} \frac{{{\text{d}}^{2} u_{{{\text{NES1}}}} }}{{{\text{d}}t^{2} }} + k_{{{\text{NES1}}}} \left[ {u_{{{\text{NES1}}}} - \sum\limits_{n = 1}^{{\text{N}}} {\varphi_{1n} \left( {x_{{{\text{NES1}}}} } \right)\frac{{{\text{d}}q_{2n} }}{{{\text{d}}t}}} } \right]^{3} \hfill \\ \begin{array}{*{20}c} {} \\ \end{array} + C_{{{\text{NES1}}}} \left[ {\frac{{{\text{d}}u_{{{\text{NES1}}}} }}{{{\text{d}}t}} - \sum\limits_{n = 1}^{{\text{N}}} {\varphi_{1n} \left( {x_{{{\text{NES1}}}} } \right)\frac{{{\text{d}}q_{1n} }}{{{\text{d}}t}}} } \right] = 0 \hfill \\ \end{gathered}$$

and

10-b $$\begin{gathered} m_{{{\text{NES2}}}} \frac{{{\text{d}}^{2} u_{{{\text{NES2}}}} }}{{{\text{d}}t^{2} }} + k_{{{\text{NES2}}}} \left[ {u_{{{\text{NES2}}}} - \sum\limits_{m = 1}^{{\text{M}}} {\varphi_{2m} \left( {x_{{{\text{NES2}}}} } \right)\frac{{{\text{d}}q_{2m} }}{{{\text{d}}t}}} } \right]^{3} \hfill \\ \begin{array}{*{20}c} {} \\ \end{array} + C_{{{\text{NES2}}}} \left[ {\frac{{{\text{d}}u_{{{\text{NES2}}}} }}{{{\text{d}}t}} - \sum\limits_{m = 1}^{{\text{M}}} {\varphi_{2m} \left( {x_{{{\text{NES2}}}} } \right)\frac{{{\text{d}}q_{2m} }}{{{\text{d}}t}}} } \right] = 0 \hfill \\ \end{gathered}$$

Longitudinal vibration responses of the double-rod system with NESs can be gained by employing numerical methods to simultaneously solve Eqs. (9) and (10). In this work, the Runge–Kutta method is used to solve the above equations.

## Numerical analysis

This section discusses the numerical results of the double-rod system with NESs. Firstly, the validity of GTM in gaining longitudinal vibration responses of the double-rod system with NESs is studied. Secondly, the impact of NESs on the dynamic responses of the double-rod is studied. Eventually, study the parameters optimization of NESs and discuss the feasibility of controlling the vibration of the double-rod system by employing NESs. Structural parameters of the double-rod system are listed as follows, *E*_1_ = *E*_2_ = 6.89 × 10^10^ Pa, *D*_1_ = 0.06 m, *D*_2_ = 0.04 m, *ρ*_1_ = *ρ*_2_ = 2800 kg/m^3^, *L*_1_ = *L*_2_ = 0.5 m, *k*_E_ = 10^4^ N/m, *C*_E_ = 2 Ns/m, *F*_E_ = 100 N, and *k*_L1_ = *k*_L2_ = *k*_R1_ = *k*_R2_ = 5 × 10^4^ N/m.

### Validity study

**Figure 2:**
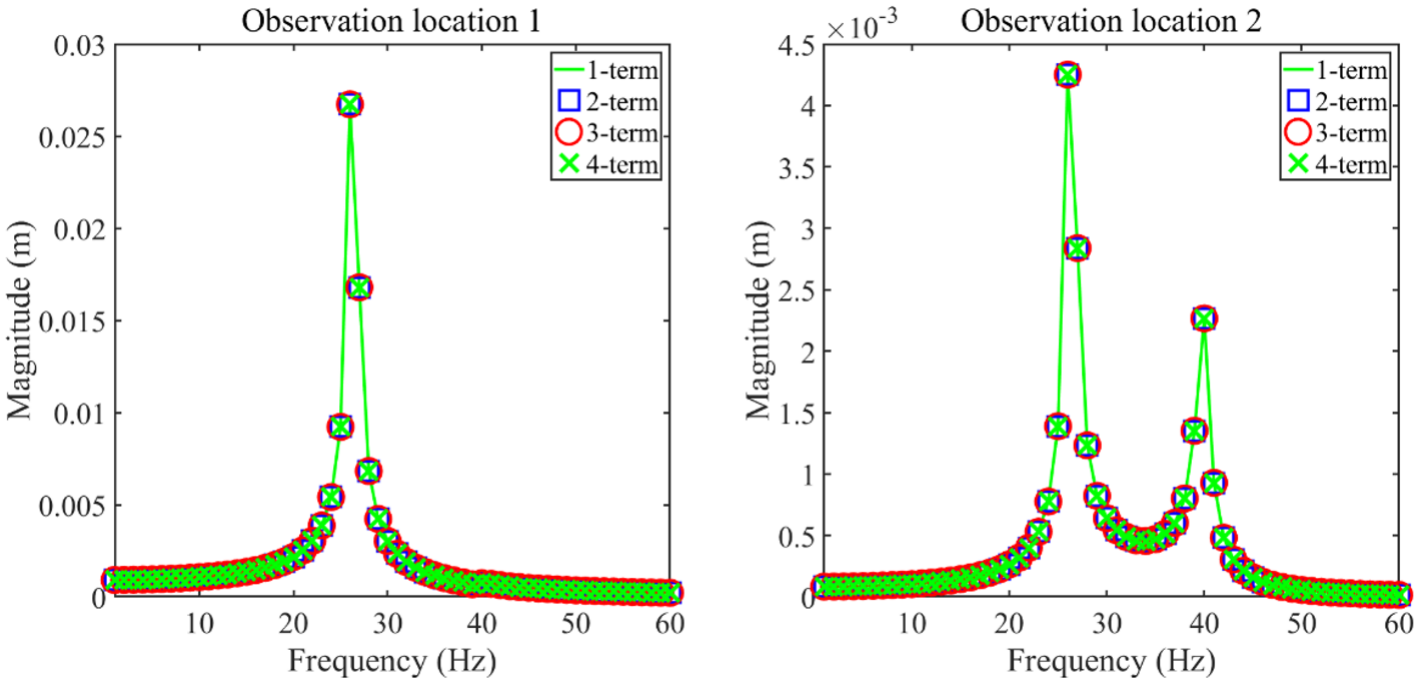
Longitudinal vibration responses under different truncation numbers.

This section studies the validity of the longitudinal vibration responses of the double-rod system with NESs gained by GTM. In this section, the parameters of NESs are listed as follows, *m*_NES1_ = *m*_NES2_ = 0.5 kg, C_NES1_ = *C*_NES2_ = 10 Ns/m, *k*_NES1_ = *k*_NES2_ = 10^6^ N/m^3^, and *x*_NES1_ = *x*_NES2_ = 0.2 m. Firstly, Fig. 2 presents longitudinal vibration responses of the double-rod system with NESs under different truncation numbers to study the stability of GTM, where observation location 1 is *x*_1_ = *L*_1_ while observation location 2 is *x*_2_ = *L*_2_. According to Fig. 2, the 1-term truncation number (N = M = 1) guarantees the stability of longitudinal vibration responses of the double-rod system with NESs. Therefore, the truncation number in the subsequent study is also set as the 1-term.

**Figure 3:**
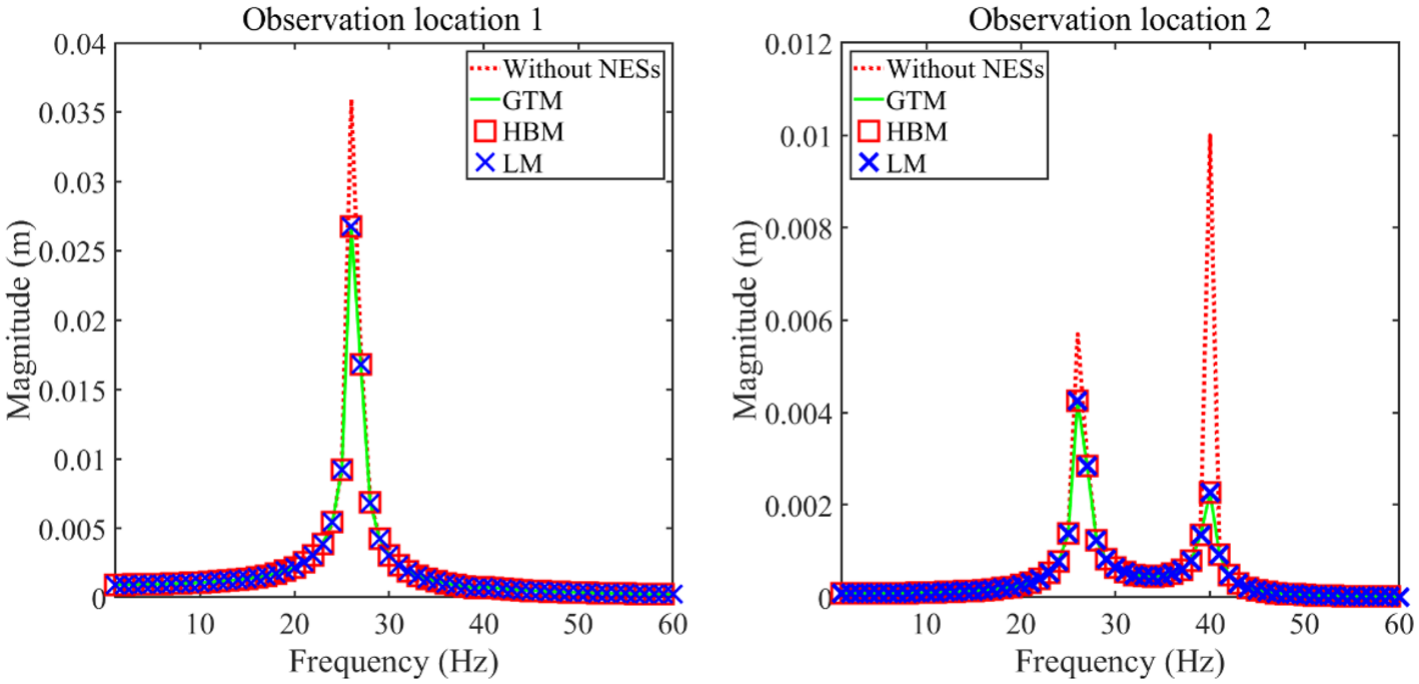
Comparison of the longitudinal vibration responses gained by various methods.

Figure 3 presents longitudinal vibration responses of the double-rod system with NESs calculated by various methods to study the correctness of GTM. From Fig. 3, longitudinal vibration responses calculated by GTM fit those calculated by the harmonic balance method (HBM) and Lagrange method (LM) smoothly, which illustrates the correctness of GTM in calculating longitudinal vibration responses of the double-rod system with NESs. Importantly, the modeling process of the LM is different from that of the GTM while the GTM and HBM calculates the vibrational displacements of the double-rod system with NESs from different domains. The detailed solution process of LM and HBM are listed in Appendices C and D. Furthermore, compared longitudinal vibration responses that consider NESs with those without NESs, find that NESs can effectively reduce vibration magnitudes of the double-rod system.

### Longitudinal frequency responses impacted by NESs

**Figure 4:**
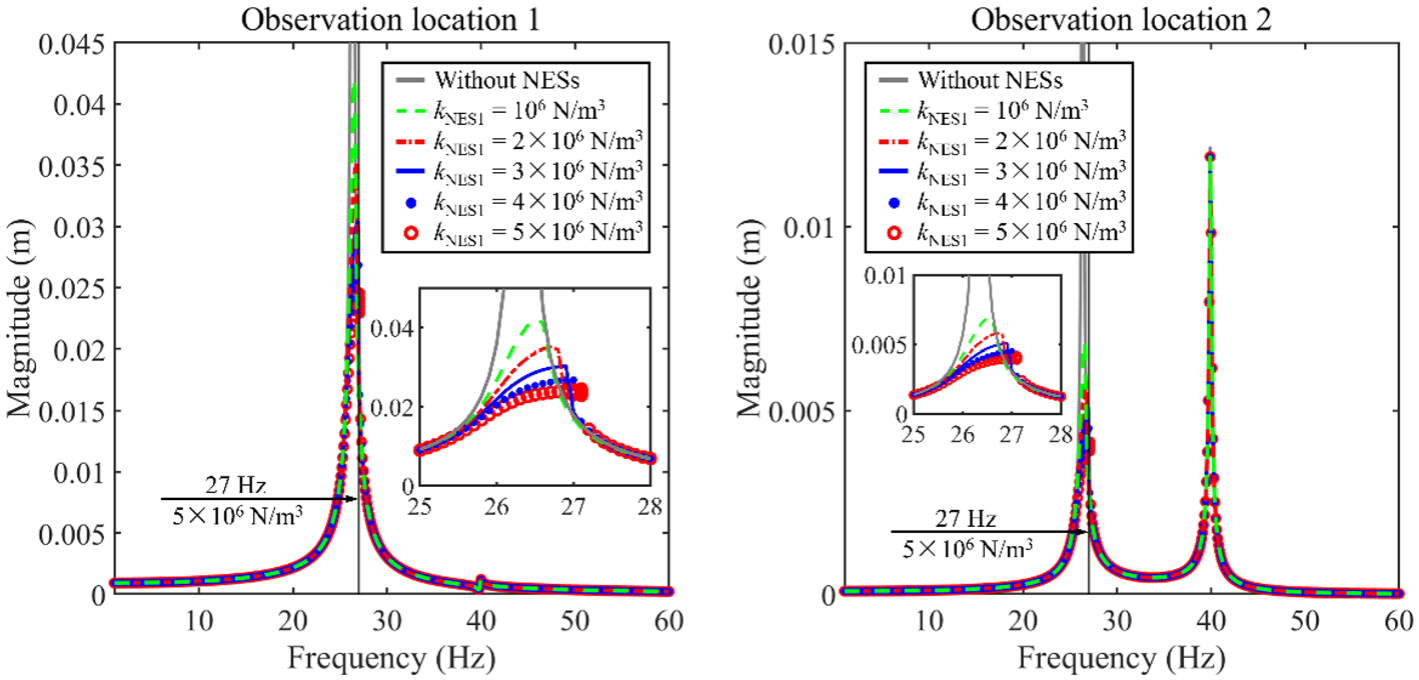
The impact of *k*_NES1_ on longitudinal frequency responses.

This section detailed studies the impact of NESs on longitudinal frequency responses of the system. Figure 4 presents the impact of *k*_NES1_ on the longitudinal frequency responses. A local subfigure close to the amplitude-frequency response resonance region is added in the revised manuscript. In this numerical example, only NES 1 acts on the double-rod system. According to Fig. 4, the parameter variation of NES 1 impacts longitudinal vibration responses of the double-rod system, obviously. Specifically, within the parameter variation range, the variation of *k*_NES1_ mainly impacts longitudinal vibration responses at the 1st primary resonance area while the variation of *k*_NES1_ rarely impacts the 2nd primary resonance area. Furthermore, the jump phenomenon appears near the 1st primary resonance area. From the aspect of vibration control, the existence of NES 1 significantly reduces peaks of magnitudes near the 1st primary resonance area. The growth of *k*_NES1_ positively impacts the vibration reduction of the 1st primary resonance area. In the vibration reduction process of the double-rod system by employing NES 1, there is no additional resonance area introduced in longitudinal vibration responses. From the aspect of vibration states, under certain values of *k*_NES1_, NES 1 transfers the vibration state of the double-rod system. In Fig. 4, longitudinal vibration responses present multiple continuous amplitudes while the excitation frequency is 27 Hz and *k*_NES1_ = 5 × 10^6^ N/m^3^. It should be noticed that the vibration state of longitudinal vibration responses that have multiple continuous amplitudes must be different from those that only have a single amplitude.

**Figure 5:**
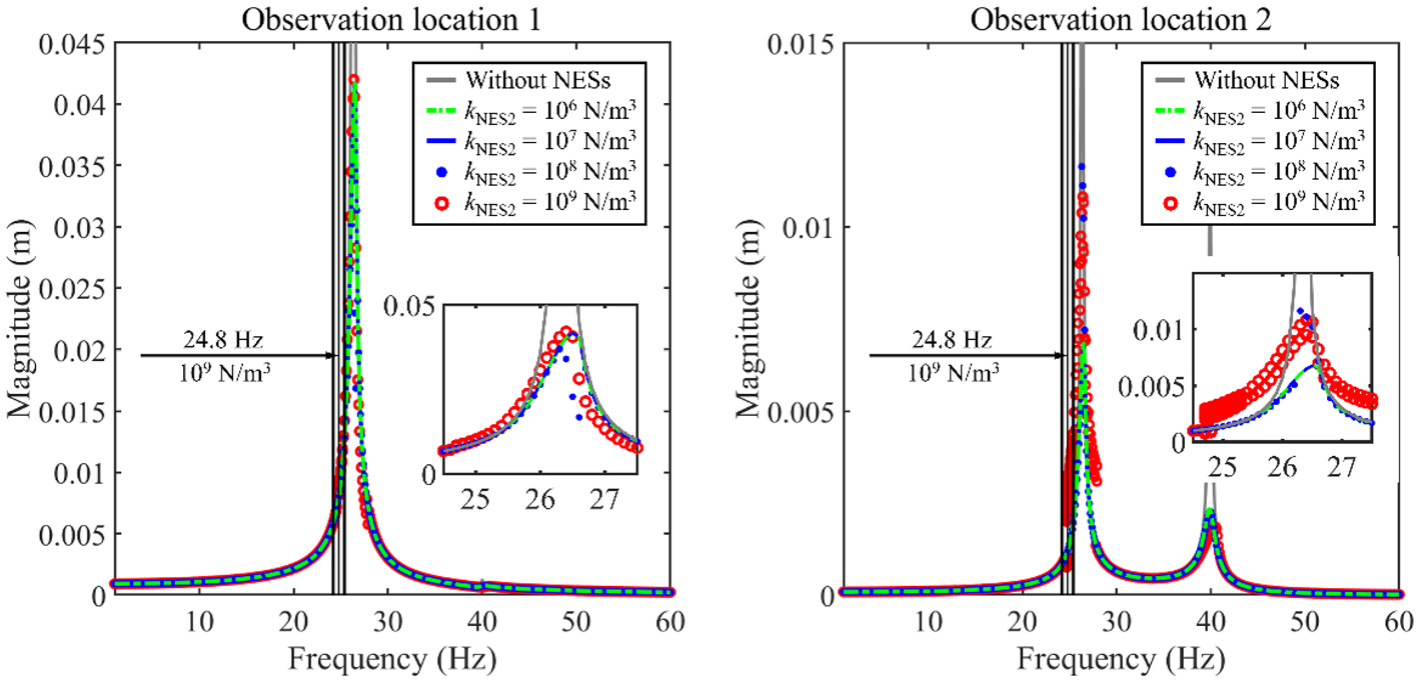
The impact of *k*_NES2_ on longitudinal frequency responses.

Figure 5 presents the impact of *k*_NES2_ on longitudinal frequency responses. In this numerical example, NES 1 and NES 2 are installed on the double-rod system. Considering the vibration magnitude of Rod 2 is less than that of Rod 1, the nonlinear force generated by NES 2 is smaller than that generated by NES 1 within the same range of parameters. To understand the impact of NES 2 on the longitudinal vibration response of the double-rod system more clearly, the parameter variation range of NES 2 in Fig. 5 is larger than that in Fig. 4. From Fig. 5, longitudinal vibration responses of the double-rod system are impacted by the parameter variation of NES 2. Specifically, within the parameter variation range, the variation of *k*_NES2_ impacts longitudinal vibration responses at the 1st and 2nd primary resonance areas greatly. From the aspect of vibration control, the existence of NES 2 significantly reduces peaks of magnitudes near the 2nd primary resonance area belonging to Rod 2. The growth of *k*_NES2_ improves the vibration reduction effectiveness of the 2nd primary resonance area belonging to Rod 2. However, the vibration reduction effectiveness of the 1st primary resonance area is weakened in the process of increasing *k*_NES2_. The above phenomenon illustrates that blindly increasing *k*_NES2_ harms the vibration reduction effectiveness for the 1st primary resonance area. The explanation for the above phenomenon is that the continuous increase of *k*_NES2_ also continuously increases the nonlinear force introduced by NES 1 when the other parameters of the double-rod system remain certain. The continuous increase of nonlinear force worsens the vibration reduction of the 1st primary resonance area. Considering the above phenomenon, it is of great significance to optimize the parameters of NESs. Furthermore, no additional resonance area is introduced in longitudinal vibration responses with the existence of NESs. From the aspect of vibration states, NES 2 changes the vibration state of the double-rod system under certain values of *k*_NES2_. In Fig. 5, longitudinal vibration responses present multiple continuous amplitudes while the excitation frequency is 24.8 Hz and *k*_NES2_ = 10^9^ N/m^3^. Similar to the analysis related to Fig. 4, the vibration state of longitudinal vibration responses that have multiple continuous amplitudes presents differences from those that only have a single amplitude.

Compared to the analysis related to Fig. 4 with that related to Fig. 5, under reasonable parameters, NES 1 can effectively reduce vibration magnitudes of the 1st primary resonance area while NES 2 is positive to the vibration reduction of the 2nd primary resonance area. The above phenomenon indicates that NES 1 and NES 2 should be simultaneously installed in the double-rod system to reduce the vibration of the first two-order primary resonance areas of the double-rod system. Meanwhile, synchronous optimizing parameters of NES 1 and NES 2 play an important role in the vibration control of the double-rod system.

**Figure 6:**
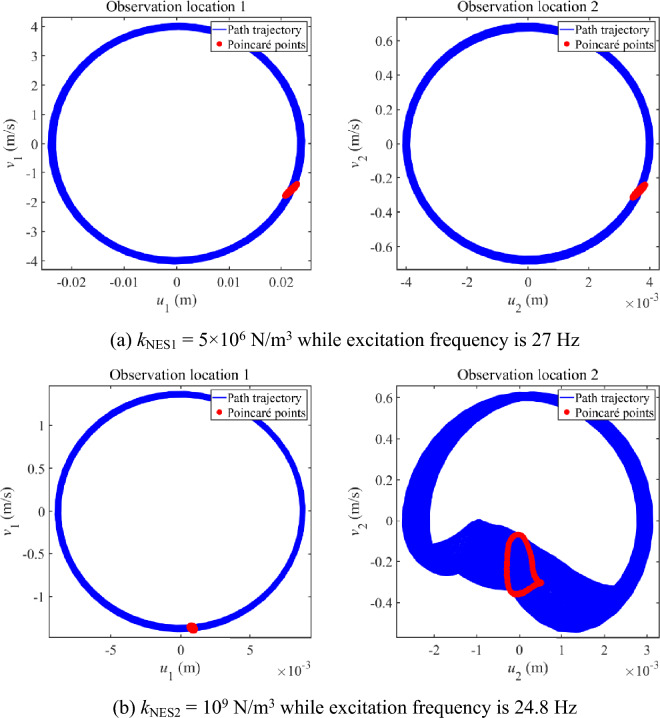
Phase trajectory and Poincaré points of longitudinal vibration responses that have multiple continuous amplitudes.

Figure 6 further studies the vibration state of longitudinal vibration responses that have multiple continuous amplitudes. Phase trajectories and Poincaré points are graphed in Fig. 6. From each subfigure in Fig. 6, phase trajectories of the observation locations remain stable and Poincaré points compose an entire closed curve. Comprehensive considering the above phenomena shown in Fig. 6, it can be concluded that the vibration state of longitudinal vibration responses that have multiple continuous amplitudes presents the quasi-periodic vibration state. It is worth mentioning that the quasi-periodic vibration state is a critical state, which is easy to develop as a chaotic state.

**Figure 7:**
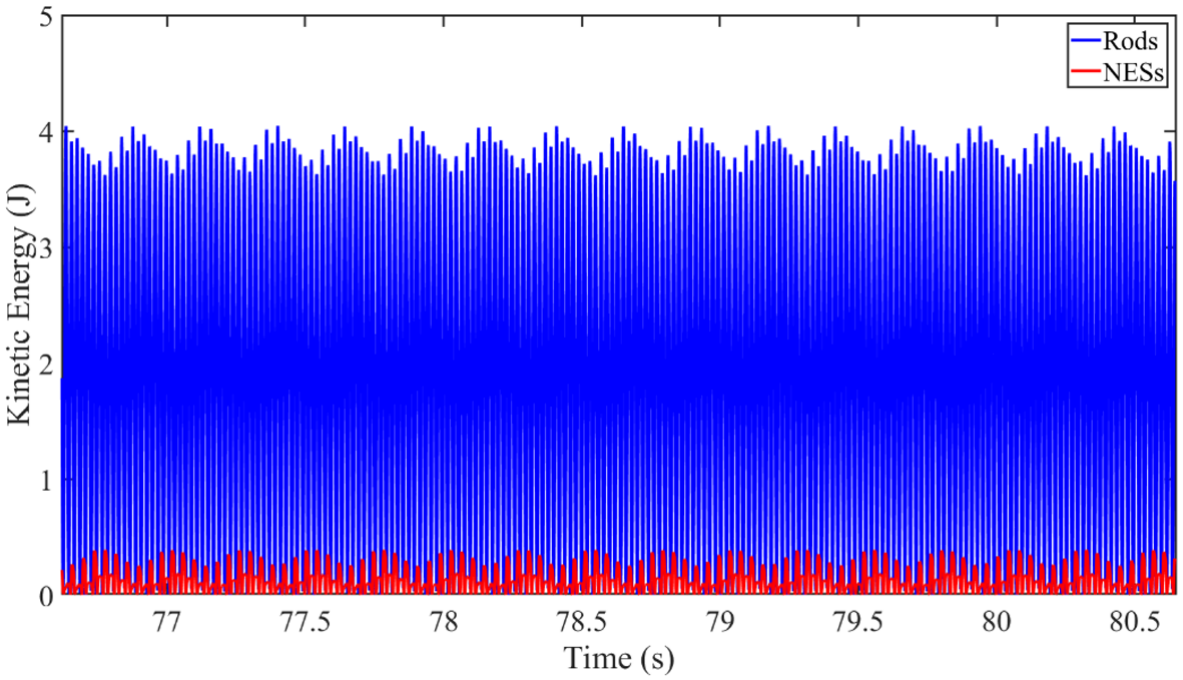
Kinetic energy of rods and NESs under complicated responses.

Figure 7 gives the vibration kinetic energy of rods and NESs under complicated response, where *k*_NES1_ = 10^6^ N/m^3^ and *k*_NES2_ = 10^9^ N/m^3^. In this numerical example, the excitation frequency is 25.1 Hz. From Fig. 7, peaks of vibration kinetic energy results belonging to rods and CNES change periodically. Worthily, the phase difference exists in the max value of vibration kinetic energy responses of rods and NESs, suggesting that vibration energy of rods targeted transferred into NESs in certain time intervals. It can be concluded that targeted energy transfer phenomenon appears under complicated responses.

### Longitudinal vibration responses under determined excitations impacted by NESs

**Figure 8:**
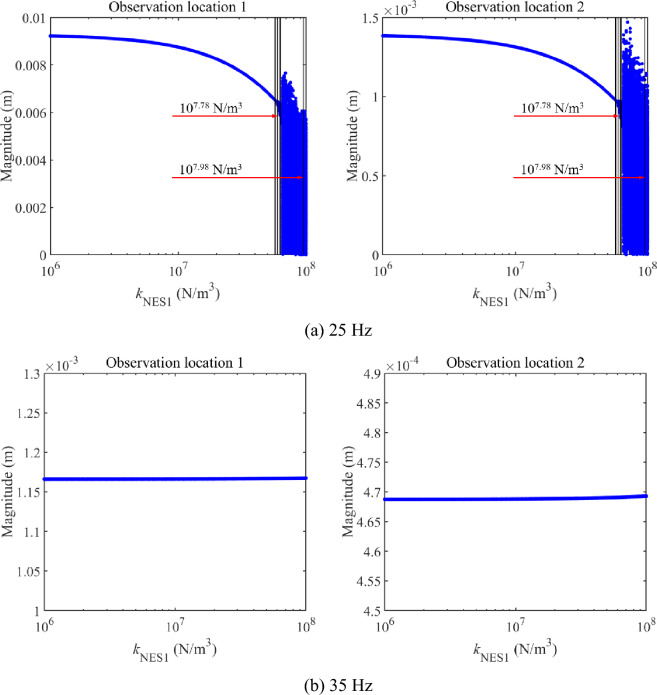
The impact of *k*_NES1_ on longitudinal vibration responses.

This section discusses the feasibility of controlling the vibration of the double-rod system under a determined excitation by employing NESs. Figure 8 gives the impact of *k*_NES1_ on longitudinal vibration responses under a determined excitation, where frequencies of the harmonic excitation are chosen as 25 Hz and 35 Hz. According to the analysis in Section “Longitudinal frequency responses impacted by NESs”, 25 Hz is in the 1st primary resonance area while 35 Hz is out of the 1st primary resonance area. From Fig. 8, changing *k*_NES1_ effectively impacts longitudinal vibration responses of the double-rod system under the excitation frequency which is near the 1st primary resonance area. In contrast, changing *k*_NES1_ has little impact on longitudinal vibration responses under the excitation frequency which is out of the 1st primary resonance area. For the longitudinal vibration responses under an excitation frequency close to the 1st primary resonance area, the growth of *k*_NES1_ is good for vibration reduction at observation locations. In the double-rod system, the continuous growth of *k*_NES1_ causes complex longitudinal vibration responses. According to the width of amplitude distribution, complex longitudinal vibration responses can be divided into two areas. The vibration state of complex longitudinal vibration responses seems to be different from the stable longitudinal vibration responses, which will be further studied in Fig. 10.

**Figure 9:**
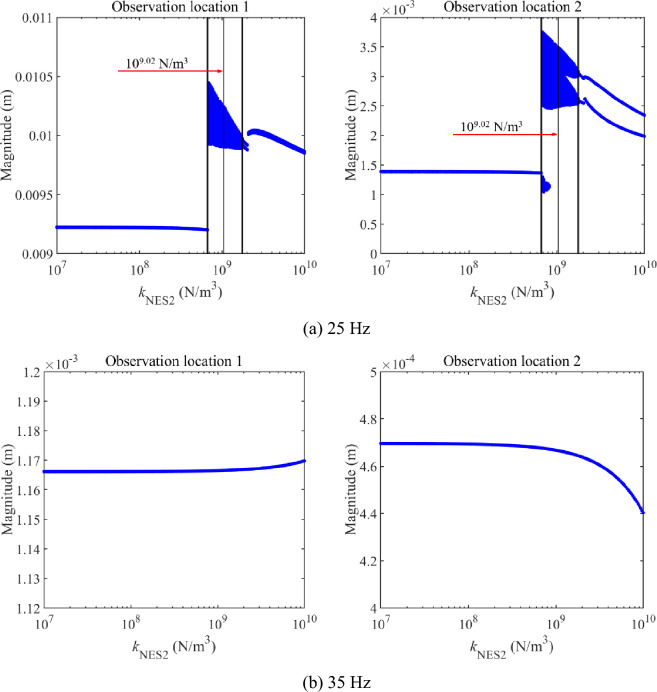
The impact of *k*_NES2_ on longitudinal vibration responses.

Similarly, Fig. 9 gives the impact of *k*_NES2_ on longitudinal vibration responses under a single-frequency excitation, where frequencies of the harmonic excitation are also chosen as 25 Hz and 35 Hz. According to Fig. 9, changing *k*_NES2_ greatly impacts longitudinal vibration responses under 25 Hz. However, changing *k*_NES2_ impacts longitudinal vibration responses under 35 Hz, weakly. For the longitudinal vibration responses under 25 Hz, magnitudes of the double-rod system keep low with the growth of *k*_NES2_ in a wide range. When *k*_NES2_ exceeds certain values, complex longitudinal vibration responses appear in Fig. 9. With the continuous growth of *k*_NES2_, complex longitudinal vibration responses gradually vanish. The explanation for the above phenomenon is listed in the following. According to Fig. 5, the growth of *k*_NES2_ impacts peaks of longitudinal vibration responses near the 1st primary resonance area. In the process of increasing *k*_NES2_, peaks of longitudinal vibration displacement under certain excitation frequencies near the 1st primary resonance area increase first and then decrease. Under certain peaks belonging to longitudinal vibration responses, complex longitudinal vibration responses appear in the double-rod system. Returning to Fig. 9, the disappearance and appearance of complex longitudinal vibration responses are the mappings of the above phenomena.

Compared to the analysis related to Fig. 8 with that related to Fig. 9, under reasonable single-frequency excitation, changing parameters of NESs can effectively change the vibration state and vibration magnitudes of the double-rod system. In the same parameter variation range of nonlinear stiffness, the change of nonlinear stiffness belonging to NES 1 can more easily influence vibration response of the double-rod system, suggesting the nonlinear stiffness sensitive region of NES 1 and NES 2 presents differences for the vibration responses of the double-rod system. The above analysis illustrates the feasibility of controlling the vibration of the double-rod system under a single-frequency excitation by utilizing NESs.

**Figure 10:**
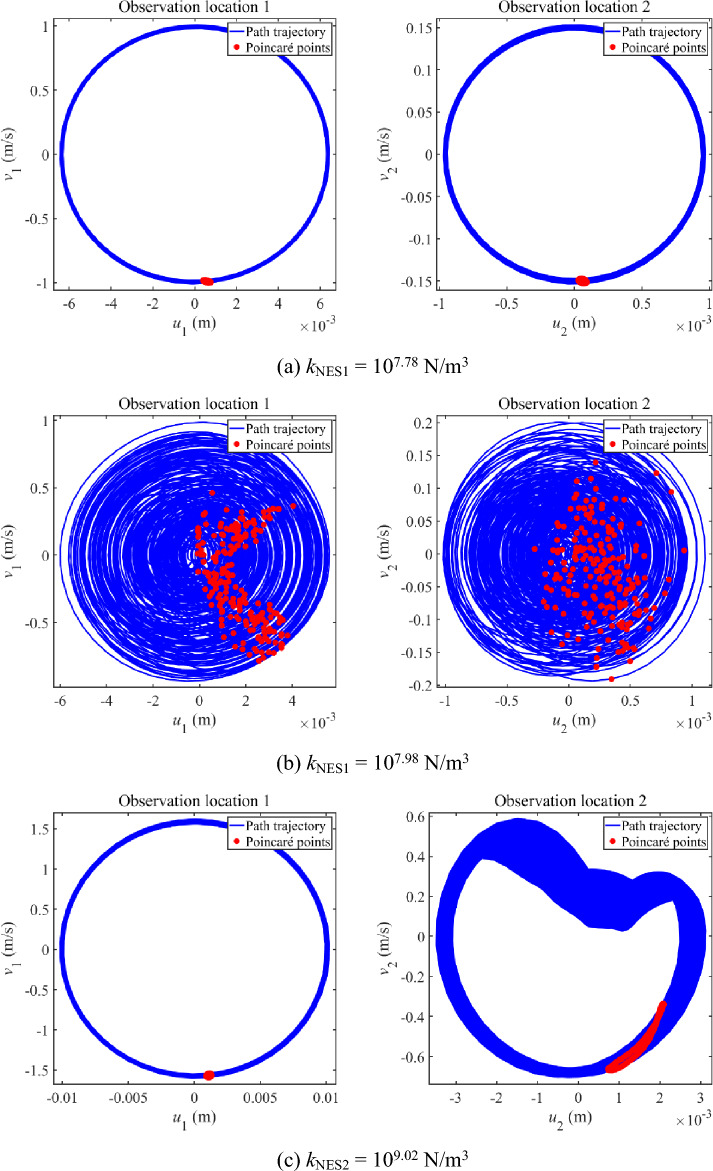
Phase trajectory and Poincaré points of complex longitudinal vibration responses under 25 Hz.

Then, Fig. 10 further studies the vibration state of complex longitudinal vibration responses shown in Figs. 8 and 9. Phase trajectories and Poincaré points are graphed in Fig. 10. From the subfigures in Fig. 10a,b, phase trajectories of the observation locations remain stable and Poincaré points compose an entire closed curve. It can be concluded that the vibration areas represented by Fig. 10a,c present the quasi-periodic vibration state. From the subfigures in Fig. 10b, phase trajectories move in a limited range while the distribution of Poincaré points seems to be disorder. It can be concluded that the vibration areas represented by Fig. 10b present the chaos vibration state.

### Parameters optimization study of NESs

Comprehensive considering the analysis in Sections “Longitudinal frequency responses impacted by NESs” and “Longitudinal vibration responses under determined excitations impacted by NESs”, reasonable parameters of NESs are good for vibration reduction of the double-rod system. Therefore, it is necessary to conduct an optimization study of NESs. This section concentrates on the parameter optimization study of NESs for longitudinal vibration responses of the double-rod system.

**Figure 11:**
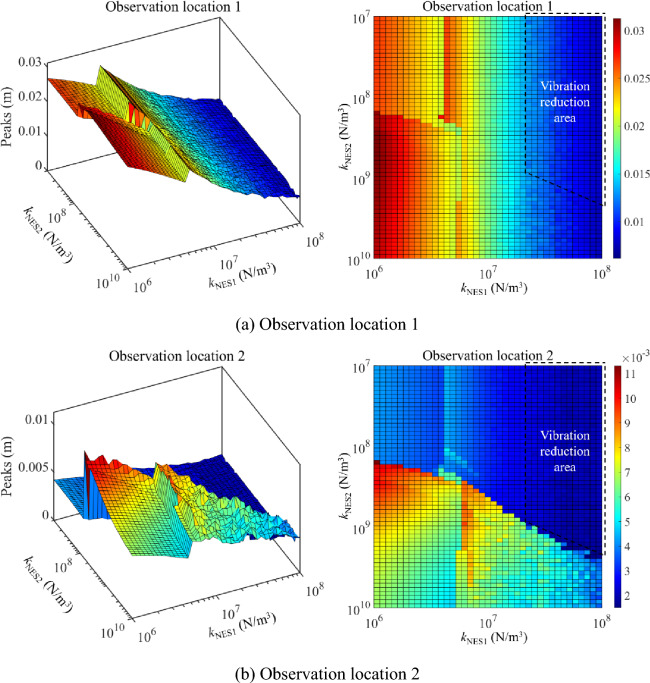
Parameter optimization (nonlinear stiffness) of NESs under the 1st primary resonance area (20–35 Hz).

Figure 11 presents the parameter optimization results of NESs under the 1st primary resonance area (20 Hz to 35 Hz), where peaks of such an area are chosen as the target function. It can be found that peaks sensitive areas related to the nonlinear stiffness of NESs occur in Fig. 11. In the sensitive areas, changing *k*_NES1_ or *k*_NES2_ can effectively impact peaks of longitudinal vibration responses in the 1st primary resonance area. Meanwhile, it can be found that peaks of the 1st primary resonance area are no longer smooth in the parameter variation range of NESs, which means complex longitudinal vibration responses of the double-rod system happen. From a global perspective, with the continuous increase of *k*_NES1_, peaks of the 1st primary resonance area belonging to Rod 1 gradually decrease. However, the change of *k*_NES2_ rarely affects peaks of 1st primary resonance area belonging to Rod 1. From the aspect of vibration reduction, the nonlinear stiffness of NESs staying in the vibration reduction area can effectively reduce the vibration of the double-rod system under the 1st primary resonance area.

**Figure 12:**
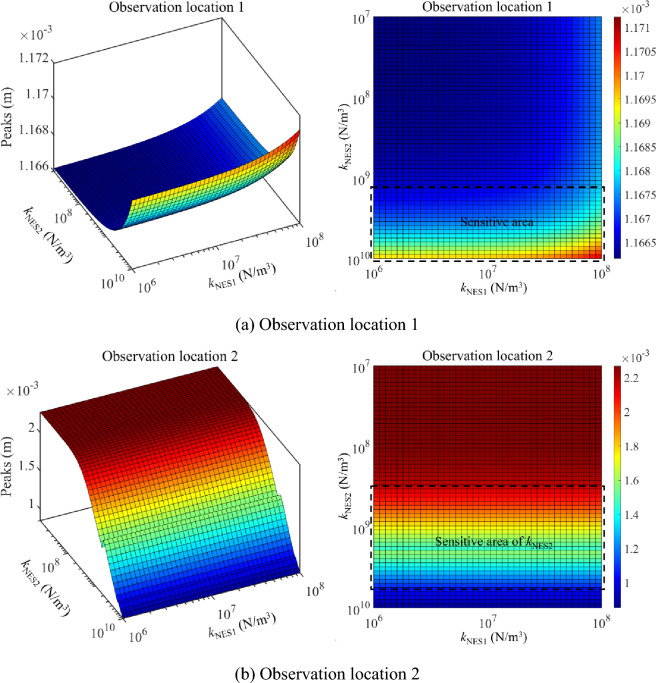
Parameter optimization (nonlinear stiffness) of NESs under the 2nd primary resonance area (35–50 Hz).

Figure 12 presents the parameter optimization results of NESs under the 2nd primary resonance area (35–50 Hz). Peaks of the above area are defined as the target function. Figure 12 shows a peaks-sensitive area related to nonlinear stiffness in the parameter optimization results. In the peaks-sensitive area, longitudinal vibration responses in the 2nd primary resonance area are easily changed with the change of *k*_NES1_ or *k*_NES2_. Peaks of the 2nd primary resonance area keep smoothly in the parameter variation range of NESs. Furthermore, it should be noticed that changing the nonlinear stiffness of NESs impacts peaks of the 2nd primary resonance area belonging to Rod 1 in a limited range. The explanation for the above phenomenon is that peaks of the 2nd primary resonance belonging to Rod 1 keep at a low level, causing the impact of nonlinear stiffness on them to be not obvious. From a global perspective, with the continuous increase of *k*_NES2_, peaks of the 2nd primary resonance area belonging to Rod 2 gradually decrease. From the aspect of vibration reduction, reasonable nonlinear stiffness of NESs has a positive impact on the vibration reduction of the vibration belonging to Rod 2 under the 2nd primary resonance area.

Compressively considering the analysis related to Figs. 11 and 12, for primary resonance areas, nonlinear stiffness belonging to NES 1 and NES 2 can both impact vibration reduction of the double-rod system under a suitable variation range. The suitable variation range of nonlinear stiffness provides the effective parameter control region of semi-active control of the double-rod system by utilizing NESs. Furthermore, reasonable nonlinear stiffness can simultaneously reduce the vibration at different primary resonance areas of the double-rod system.

**Figure 13:**
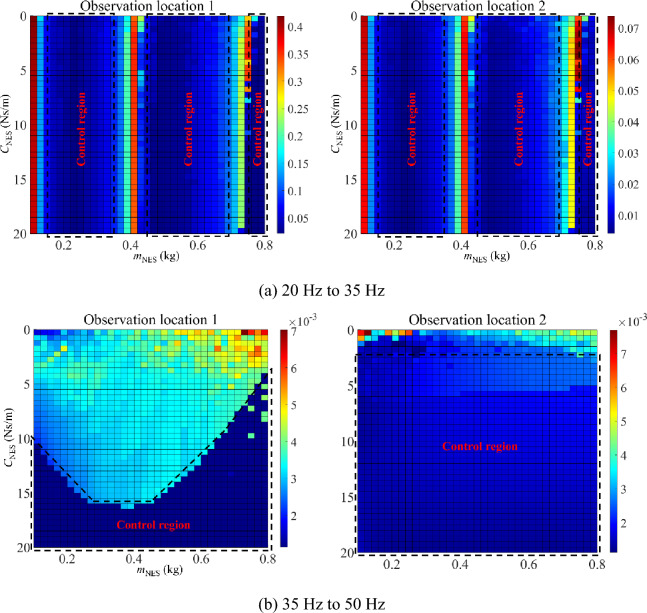
Parameter optimization (motion mass and viscous damping) of NESs under the resonance areas.

Figure 13 presents the parameter optimization results (motion mass and external viscous damping) of NESs under resonance areas, where peaks of such an area are chosen as the target function. In this numerical example, nonlinear stiffness of NESs is both set as 10^9^ N/m^3^. Figure 13a corresponds to the 1st primary resonance area (20–35 Hz) while Fig. 13b corresponds to the 2nd primary resonance area (35–50 Hz). For the first two resonance areas, there are several control regions appear in optimized results, where magnitudes of the double-rod system with NESs can be controlled in an ideal level under each control region. Furthermore, under certain combinations of motion mass and external viscous damping, changing values of each parameter belonging to NESs sensitively influences magnitudes of the double-rod system, providing a parameter adjustable range for the double-rod system with NESs. On the whole, reasonable values of motion mass and external viscous damping can effectively reduce the vibration of the double-rod system under resonance areas.

**Figure 14:**
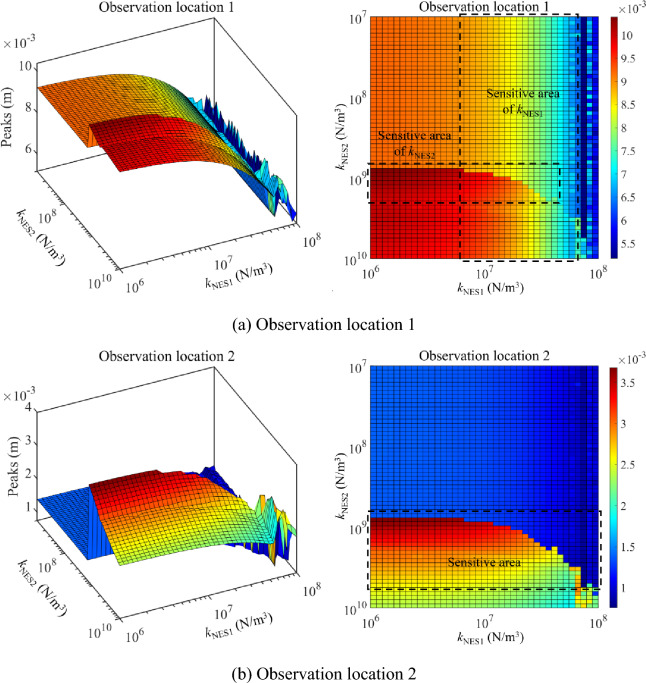
Parameter optimization of NESs under 25 Hz.

Then, the parameter optimization study of NESs under the single-frequency excitation is studied. In this numerical example, excitation frequencies are chosen as 25 Hz and 35 Hz, where 25 Hz is located at the 1st primary resonance area while 35 Hz is out of the primary resonance area. Figure 14 presents the parameter optimization results of NESs under 25 Hz. From Fig. 14, sensitive areas related to the nonlinear stiffness of NESs appear in the parameter results. It can be observed that peaks of the longitudinal vibration responses under a single-frequency excitation are no longer smooth in the parameter variation range of NESs, suggesting complex longitudinal vibration responses of the double-rod system appear. From the aspect of vibration reduction, reasonable parameters of NESs effectively reduce peaks of the longitudinal vibration responses under a single-frequency excitation. Meanwhile, peaks of longitudinal vibration responses change greatly in the variation range of nonlinear stiffness. The above phenomenon means that it is necessary to optimize the nonlinear stiffness of NESs for longitudinal vibration responses under the excitation frequency which is near the primary resonance area.

**Figure 15:**
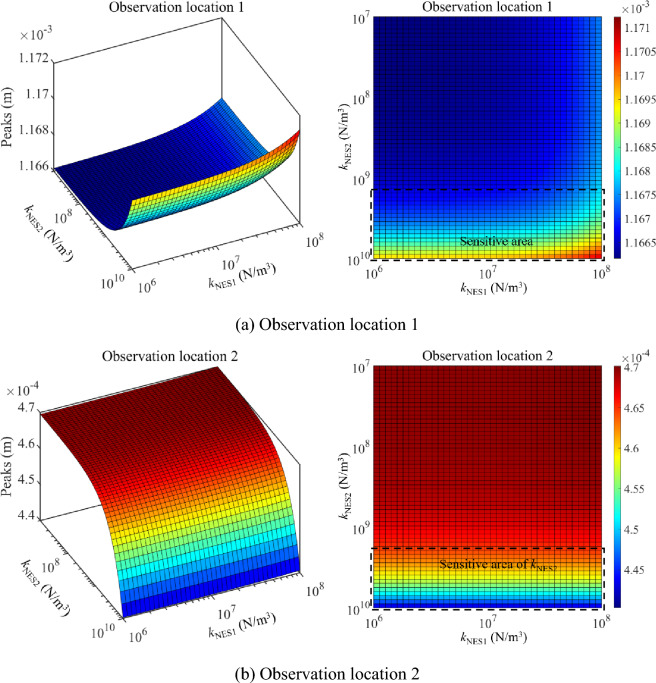
Parameter optimization of NESs under 35 Hz.

Figure 15 presents the parameter optimization results of NESs under 35 Hz. From Fig. 15, peaks-sensitive areas related to the nonlinear stiffness of NESs appear in the parameter results. However, peaks are impacted slightly by the change of nonlinear stiffness related to NESs. Meanwhile, peaks of the longitudinal vibration responses under 35 Hz stay at a low level for the whole variation range of nonlinear stiffness. The above phenomenon suggests it is unnecessary to optimize the nonlinear stiffness of NESs for longitudinal vibration responses under 35 Hz. Compressively considering the analysis related to Figs. 14 and 15, for the single-frequency excitation near the primary resonance area, optimizing the nonlinear stiffness of NESs is positive for the vibration reduction of the system. However, for the single-frequency excitation out of the primary resonance area, it is unnecessary to optimize the nonlinear stiffness of NESs for longitudinal vibration responses.

**Figure 16:**
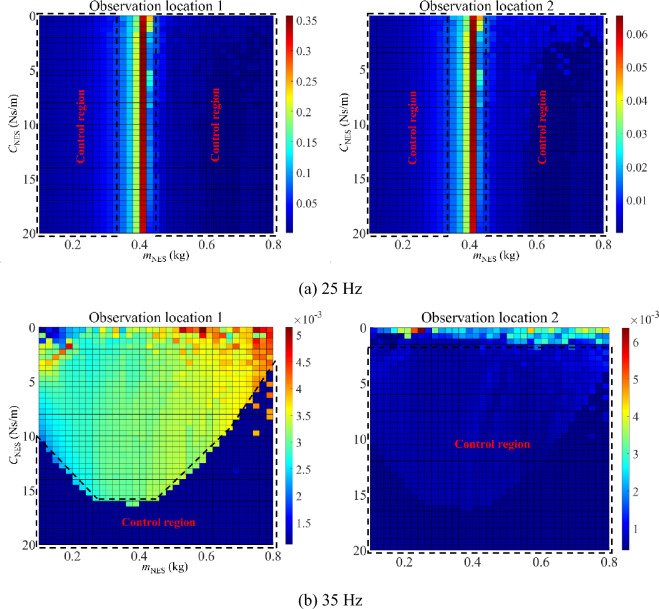
Parameter optimization of NESs under single-frequency excitation.

Figure 16 presents the parameter optimization results (motion mass and external viscous damping) of NESs under single-frequency excitation, where peaks of such an area are chosen as the target function. In this numerical example, nonlinear stiffness of NESs is both set as 10^9^ N/m^3^. Figure 16a corresponds to the 25 Hz while Fig. 16b corresponds to the 35 Hz. For the single-frequency excitations, several control regions appear in optimized results, where magnitudes of the double-rod system with NESs can be controlled effectively in such regions. Meanwhile, under certain combinations of motion mass and external viscous damping, changing values of each parameter belonging to NESs has a great influence on magnitudes of the double-rod system, providing a parameter adjustable range for the double-rod system with NESs under single-frequency excitations. On the whole, reasonable values of motion mass and external viscous damping can effectively reduce the vibration of the double-rod system under single-frequency excitations.

## Conclusion

This study introduces a predictive model for longitudinal vibrations in a double-rod system with NESs. The generalized Hamilton principle is employed to derive governing equations of the double-rod system. Subsequently, the longitudinal vibration responses of the double-rod system are numerically determined utilizing GTM method. Following the validation of the numerical results, this study examines the longitudinal vibration responses of a double-rod system subjected to NESs. After guaranteeing the validity of the numerical results, longitudinal vibration responses of the double-rod system affected by NESs are discussed. Conclusions can be drawn based on the following results:

1. A vibration prediction model for the double-rod system with NESs is proposed and validated. The selection of an appropriate truncation number is essential to ensure the stability of longitudinal vibration responses in the system.
2. The longitudinal frequency responses of the double-rod system are impacted by NESs. While the vibration of the double-rod system can be mitigated by the application of NES 1 solely on Rod 1, the concurrent installation of NES 1 and NES 2 on the double-rod system can more efficiently reduce the vibration within the first two-order primary resonance regions of the double-rod system.
3. The longitudinal single-frequency responses of the double-rod system are impacted by NESs. Under reasonable single-frequency excitations, modifying the parameters of NESs can significantly alter the vibration state and magnitudes of the double-rod system. The nonlinear stiffness sensitive region of NES 1 and NES 2 exhibits variations in the vibration responses of the double-rod system.
4. Synchronously optimizing the parameters of NES 1 and NES 2 is crucial for vibration control in the double-rod system. The sensitive parameter regions of NESs offer the potential to regulate the vibration of the double-rod system through the utilization of NESs.

### Supplementary Information

Supplementary Information.

## Data Availability

The datasets generated during and/or analyzed during the current study are available from the corresponding author upon reasonable request.
