# Supplementary material for: Longitudinal vibration control of a double-rod system by employing nonlinear energy sinks
Source: Sci Rep. 2024 Apr 20;14:9046. doi: 10.1038/s41598-024-59644-w (PMC11031570; doi:10.1038/s41598-024-59644-w)
Supplement: Supplementary file 1 — Supplementary Information. [file 41598_2024_59644_MOESM1_ESM.docx]

# APPENDIX A

$V_{\text{Rod1}}=\frac{1}{2}E_{1}S_{1}\int_{0}^{L_{1}} \left(\frac{\partial u_{1}}{\partial x_{1}}\right)^{2}\text{d}x_{1}$ (A-1)

$V_{\text{Rod2}}=\frac{1}{2}E_{2}S_{2}\int_{0}^{L_{2}} \left(\frac{\partial u_{2}}{\partial x_{2}}\right)^{2}\text{d}x_{2}$ (A-2)

$V_{\text{Boundary1}}=\frac{1}{2}k_{\text{L1}}u_{1}^{2}\left(0,t\right)+\frac{1}{2}k_{\text{R1}}u_{1}^{2}\left(L_{1},t\right)$ (A-3)

$V_{\text{Boundary2}}=\frac{1}{2}k_{\text{L2}}u_{2}^{2}\left(0,t\right)+\frac{1}{2}k_{\text{R2}}u_{2}^{2}\left(L_{2},t\right)$ (A-4)

$V_{\text{LE}}=\int_{0}^{L_{2}} \int_{0}^{L_{1}} \delta\left(x_{1}-L_{1}\right)\delta\left(x_{2}\right)\frac{1}{2}k_{\text{E}}\left(u_{1}-u_{2}\right)^{2}\text{d}x_{1}\text{d}x_{2}$ (A-5)

$V_{\text{NES1}}=\int_{0}^{L_{1}} \delta\left(x_{1}-x_{\text{NES1}}\right)\frac{1}{4}k_{\text{NES1}}\left(u_{\text{NES1}}-u_{1}\right)^{4}\text{d}x_{1}$ (A-6)

$V_{\text{NES2}}=\int_{0}^{L_{2}} \delta\left(x_{2}-x_{\text{NES2}}\right)\frac{1}{4}k_{\text{NES2}}\left(u_{\text{NES2}}-u_{2}\right)^{4}\text{d}x_{2}$ (A-7)

$T_{\text{Rod1}}=\frac{1}{2}\rho_{1}S_{1}\int_{0}^{L_{1}} \left(\frac{\partial u_{1}}{\partial t}\right)^{2}\text{d}x_{1}$ (A-8)

$T_{\text{Rod2}}=\frac{1}{2}\rho_{2}S_{2}\int_{0}^{L_{2}} \left(\frac{\partial u_{2}}{\partial t}\right)^{2}\text{d}x_{2}$ (A-9)

$T_{\text{NES1}}=\frac{1}{2}m_{\text{NES1}}\left(\frac{\text{d}u_{\text{NES1}}}{\text{d}t}\right)^{2}$ (A-10)

$T_{\text{NES2}}=\frac{1}{2}m_{\text{NES2}}\left(\frac{\text{d}u_{\text{NES2}}}{\text{d}t}\right)^{2}$ (A-11)

$\delta W_{\text{E}}=\int_{0}^{L_{1}} \delta\left(x_{1}\right)F_{\text{E}}\sin\left(\omega t\right)\delta u_{1}\text{d}x_{1}$ (A-12)

$\delta W_{\text{LE}}=-\int_{0}^{L_{2}} \int_{0}^{L_{1}} \delta\left(x_{1}-L_{1}\right)\delta\left(x_{2}\right)C_{\text{E}}\left[\frac{\partial u_{1}}{\partial t}-\frac{\partial u_{2}}{\partial t}\right]\delta\left(u_{1}-u_{2}\right)\text{d}x_{1}\text{d}x_{2}$ (A-13)

$\delta W_{\text{NES1}}=-\int_{0}^{L_{1}} \delta\left(x_{1}-x_{\text{NES1}}\right)C_{\text{NES1}}\left(\frac{\partial u_{1}}{\partial t}-\frac{\text{d}u_{\text{NES1}}}{\text{d}t}\right)\delta\left(u_{1}-u_{\text{NES1}}\right)\text{d}x_{1}$ (A-14)

$\delta W_{\text{NES2}}=-\int_{0}^{L_{2}} \delta\left(x_{2}-x_{\text{NES2}}\right)C_{\text{NES2}}\left(\frac{\partial u_{2}}{\partial t}-\frac{\text{d}u_{\text{NES2}}}{\text{d}t}\right)\delta\left(u_{2}-u_{\text{NES2}}\right)\text{d}x_{2}$ (A-15)

# APPENDIX B

Governing equations of rods as well as motion equations of NESs can be derived by putting Eqs. (B-1) to (B-15) into Eq. (4).

$\int_{t_{1}}^{t_{2}} \delta V_{\text{Rod1}}dt=\int_{t_{1}}^{t_{2}} \left(\left.E_{1}S_{1}\frac{\partial u_{1}}{\partial x_{1}}\right|_{x_{1}=L_{1}}-\left.E_{1}S_{1}\frac{\partial u_{1}}{\partial x_{1}}\right|_{x_{1}=0}\right)\delta u_{1}\text{d}t-\int_{t_{1}}^{t_{2}} \int_{0}^{L_{1}} E_{1}S_{1}\frac{\partial^{2}u_{1}}{\partial x_{1}^{2}}\text{d}x_{1}\delta u_{1}\text{d}t$ (B-1)

$\int_{t_{1}}^{t_{2}} \delta V_{\text{Rod2}}dt=\int_{t_{1}}^{t_{2}} \left(\left.E_{2}S_{2}\frac{\partial u_{2}}{\partial x_{2}}\right|_{x_{2}=L_{2}}-\left.E_{2}S_{2}\frac{\partial u_{2}}{\partial x_{2}}\right|_{x_{2}=0}\right)\delta u_{2}\text{d}t-\int_{t_{1}}^{t_{2}} \int_{0}^{L_{2}} E_{2}S_{2}\frac{\partial^{2}u_{2}}{\partial x_{2}^{2}}\text{d}x_{2}\delta u_{2}\text{d}t$(B-2)

$\int_{t_{1}}^{t_{2}} \delta V_{\text{Boundary1}}\text{d}t=\int_{t_{1}}^{t_{2}} \left[k_{\text{L1}}u_{1}\left(0,t\right)+k_{\text{R1}}u_{1}\left(L_{1},t\right)\right]\delta u_{1}\text{d}t$ (B-3)

$\int_{t_{1}}^{t_{2}} \delta V_{\text{Boundary2}}\text{d}t=\int_{t_{1}}^{t_{2}} \left[k_{\text{L2}}u_{2}\left(0,t\right)+k_{\text{R2}}u_{2}\left(L_{2},t\right)\right]\delta u_{2}\text{d}t$ (B-4)

$\begin{matrix} \int_{t_{1}}^{t_{2}} \delta V_{\text{LE}}\text{d}t=\int_{t_{1}}^{t_{2}} \delta\left(x_{1}-L_{1}\right)k_{\text{E}}\left[u_{1}-u_{2}\left(0,t\right)\right]\delta u_{1}\text{d}t \\ \begin{matrix} & & & +\int_{t_{1}}^{t_{2}} \delta\left(x_{2}\right)k_{\text{E}}\left[u_{2}-u_{1}\left(L_{1},t\right)\right]\delta u_{2}\text{d}t \end{matrix} \end{matrix}$ (B-5)

$\begin{matrix} \int_{t_{1}}^{t_{2}} \delta V_{\text{NES1}}\text{d}t=\int_{t_{1}}^{t_{2}} k_{\text{NES1}}\left[u_{\text{NES1}}-\delta\left(x_{1}-x_{\text{NES1}}\right)u_{1}\right]^{3}\delta u_{\text{NES1}}\text{d}t \\ \begin{matrix} & & & +\int_{t_{1}}^{t_{2}} \int_{0}^{L_{1}} \delta\left(x_{1}-x_{\text{NES1}}\right)k_{\text{NES1}}\left(u_{1}-u_{\text{NES1}}\right)^{3}\delta u_{1}\text{d}x_{1}\text{d}t \end{matrix} \end{matrix}$ (B-6)

$\begin{matrix} \int_{t_{1}}^{t_{2}} \delta V_{\text{NES2}}\text{d}t=\int_{t_{1}}^{t_{2}} k_{\text{NES2}}\left[u_{\text{NES2}}-\delta\left(x_{2}-x_{\text{NES2}}\right)u_{2}\right]^{3}\delta u_{\text{NES2}}\text{d}t \\ \begin{matrix} & & & \end{matrix}+\int_{t_{1}}^{t_{2}} \int_{0}^{L_{2}} \delta\left(x_{2}-x_{\text{NES2}}\right)k_{\text{NES2}}\left(u_{2}-u_{\text{NES2}}\right)^{3}\delta u_{2}\text{d}x_{2}\text{d}t \end{matrix}$ (B-7)

$\int_{t_{1}}^{t_{2}} \delta T_{\text{Rod1}}\text{d}t=-\int_{t_{1}}^{t_{2}} \int_{0}^{L_{1}} \rho_{1}S_{1}\frac{\partial^{2}u_{1}}{\partial t^{2}}\text{d}x_{1}\delta u_{1}\text{d}t$ (B-8)

$\int_{t_{1}}^{t_{2}} \delta T_{\text{Rod2}}\text{d}t=-\int_{t_{1}}^{t_{2}} \int_{0}^{L_{2}} \rho_{2}S_{2}\frac{\partial^{2}u_{2}}{\partial t^{2}}\text{d}x_{2}\delta u_{2}\text{d}t$ (B-9)

$\int_{t_{1}}^{t_{2}} \delta T_{\text{NES1}}\text{d}t=-\int_{t_{1}}^{t_{2}} m_{\text{NES1}}\frac{\text{d}^{2}u_{\text{NES1}}}{\text{d}t^{2}}\delta u_{\text{NES1}}\text{d}t$ (B-10)

$\int_{t_{1}}^{t_{2}} \delta T_{\text{NES2}}\text{d}t=-\int_{t_{1}}^{t_{2}} m_{\text{NES2}}\frac{\text{d}^{2}u_{\text{NES2}}}{\text{d}t^{2}}\delta u_{\text{NES2}}\text{d}t$ (B-11)

$\int_{t_{1}}^{t_{2}} \delta W_{\text{E}}\text{d}t=\int_{0}^{L_{1}} \delta\left(x_{1}\right)F_{\text{E}}\sin\left(\omega t\right)\text{d}x_{1}\delta u_{1}\text{d}t$ (B-12)

$\begin{matrix} \int_{t_{1}}^{t_{2}} \delta W_{\text{LE}}\text{d}t=-\int_{t_{1}}^{t_{2}} \int_{0}^{L_{2}} \int_{0}^{L_{1}} \delta\left(x_{1}-L_{1}\right)\delta\left(x_{2}\right)C_{\text{E}}\left[\frac{\partial u_{1}}{\partial t}-\frac{\partial u_{2}}{\partial t}\right]\text{d}x_{1}\text{d}x_{2}\delta u_{1}\text{d}t \\ \begin{matrix} & & & -\int_{t_{1}}^{t_{2}} \int_{0}^{L_{2}} \int_{0}^{L_{1}} \delta\left(x_{1}-L_{1}\right)\delta\left(x_{2}\right)C_{\text{E}}\left[\frac{\partial u_{2}}{\partial t}-\frac{\partial u_{1}}{\partial t}\right]\text{d}x_{1}\text{d}x_{2}\delta u_{2}\text{d}t \end{matrix} \end{matrix}$ (B-13)

$\begin{matrix} \int_{t_{1}}^{t_{2}} \delta W_{\text{NES1}}\text{d}t=-\int_{t_{1}}^{t_{2}} C_{\text{NES1}}\left[\frac{\text{d}u_{\text{NES1}}}{\text{d}t}-\delta\left(x_{1}-x_{\text{NES1}}\right)\frac{\partial u_{1}}{\partial t}\right]\delta u_{\text{NES1}}\text{d}t \\ \begin{matrix} & & & \end{matrix}-\int_{t_{1}}^{t_{2}} \int_{0}^{L_{1}} \delta\left(x_{1}-x_{\text{NES1}}\right)C_{\text{NES1}}\left(\frac{\partial u_{1}}{\partial t}-\frac{\text{d}u_{\text{NES1}}}{\text{d}t}\right)\delta u_{1}\text{d}x_{1}\text{d}t \end{matrix}$ (B-14)

$\begin{matrix} \int_{t_{1}}^{t_{2}} \delta W_{\text{NES2}}\text{d}t=-\int_{t_{1}}^{t_{2}} C_{\text{NES2}}\left[\frac{\text{d}u_{\text{NES2}}}{\text{d}t}-\delta\left(x_{2}-x_{\text{NES2}}\right)\frac{\partial u_{2}}{\partial t}\right]\delta u_{\text{NES2}}\text{d}t \\ \begin{matrix} & & & \end{matrix}-\int_{t_{1}}^{t_{2}} \int_{0}^{L_{2}} \delta\left(x_{2}-x_{\text{NES2}}\right)C_{\text{NES2}}\left(\frac{\partial u_{2}}{\partial t}-\frac{\text{d}u_{\text{NES2}}}{\text{d}t}\right)\delta u_{2}\text{d}x_{2}\text{d}t \end{matrix}$ (B-15)

# APPENDIX C

In LM, the Lagrange term of the double-rod system with NESs is derived as,

$L_{\text{System}}=T_{\text{System}}-V_{\text{System}}$ (C-1)

The vibrational displacements of the double-rod system with NESs are reformed into specific terms,

 (C-2)

and

 (C-3)

where each term in Eqs. (C-2) and (C-3) are listed as,

 (C-4)

 (C-5)

$\mathbf{q}_{1}=\left[\begin{matrix} q_{11} & ... & q_{1n} & ... & q_{\text{1N}} \end{matrix}\right]^{\text{T}}$ (C-6)

and

$\mathbf{q}_{2}=\left[\begin{matrix} q_{21} & ... & q_{2m} & ... & q_{\text{2M}} \end{matrix}\right]^{\text{T}}$ (C-7)

To ensure the uniformity of the solution procedure, **q**_3_ is defined as follows,

$\mathbf{q}_{3}=\left[\begin{matrix} u_{\text{NES1}} & u_{\text{NES2}} \end{matrix}\right]$ (C-8)

By substituting Eqs. (C-2) and (C-3), and (C-8) into Eqs. (9) and (10), proceeding with the subsequent step,

 (C-9)

where **Q***_i_* is the generalized force. Then, the Lagrange function of the double-rod system with NESs can be established. By solving the Lagrange function, the vibrational displacements of double-rod system with NESs can be obtained.

# APPENDIX D

In HBM, the aimed functions of the double-rod system with NESs are Eqs. (9) and (10), where the unknown time terms are assumed as,

$q_{1n}\left(t\right)=B_{1n}\sin\left(\omega t\right)+B_{2n}\cos\left(\omega t\right)+C_{1n}\sin\left(3\omega t\right)+C_{2n}\cos\left(3\omega t\right)$ (D-1)

and

$q_{2m}\left(t\right)=E_{1m}\sin\left(\omega t\right)+E_{2m}\cos\left(\omega t\right)+G_{1m}\sin\left(3\omega t\right)+G_{2m}\cos\left(3\omega t\right)$ (D-2)

Displacement of nonlinear couplers is assumed as,

$u_{\text{NES1}}\left(t\right)=H_{11}\sin\left(\omega t\right)+H_{21}\cos\left(\omega t\right)+J_{11}\sin\left(3\omega t\right)+J_{21}\cos\left(3\omega t\right)$ (D-3)

and

$u_{\text{NES2}}\left(t\right)=H_{12}\sin\left(\omega t\right)+H_{22}\cos\left(\omega t\right)+J_{12}\sin\left(3\omega t\right)+J_{22}\cos\left(3\omega t\right)$ (D-4)

where *B*_1_*_n_*, *B*_2_*_n_*, *C*_1_*_n_*, *C*_2_*_n_*, *E*_1_*_m_*, *E*_2_*_m_*, *G*_1_*_m_*, *G*_2_*_m_*, *H*_11_, *H*_21_, *J*_11_, *J*_21_ *H*_12_, *H*_22_, *J*_12_, and *J*_22_ are the unknown coefficients. Substituting Eqs. (D-1), (D-2), (D-3), and (D-4) into Eqs. (9) and (10), the functions can be then obtained by arranging terms related to sin(*ωt*), cos(*ωt*), sin(3*ωt*), and cos(3*ωt*). By solving the above functions, the vibrational displacements of double-rod system with NESs can be obtained by HBM.
